# Impact of Observation on Disposition of Elderly Patients Presenting to Emergency Departments with Non-Specific Complaints

**DOI:** 10.1371/journal.pone.0098097

**Published:** 2014-05-28

**Authors:** Franziska Misch, Anna Sarah Messmer, Christian Hans Nickel, Madleina Gujan, Andreas Graber, Katharina Blume, Roland Bingisser

**Affiliations:** Emergency Department, University Hospital, Basel, Switzerland; University of Glasgow, United Kingdom

## Abstract

**Background:**

Emergency Departments (EDs) have to cope with an increasing number of elderly patients, often presenting with non-specific complaints (NSC), such as generalized weakness. Acute morbidity requiring early intervention is present in the majority of patients with NSC. Therefore, an early and optimal disposition plan is crucial. The objective of this study was to prospectively study the disposition process of patients presenting to the ED with NSC.

**Methods:**

For two years, all patients presenting with NSC presenting to an urban ED were screened and consecutively included. The initial disposition plan was compared to the effective transfer after observation. Optimal disposition was defined as a high accuracy regarding disposition of patients with acute morbidity to an internal medicine ward.

**Results:**

The final study population consisted of 669 patients with NSC. Admission to internal medicine increased from 297 (44%) planned admissions to 388 (58%) effective admissions after observation. Conversely, transfers to geriatric community hospitals and discharges decreased from the initially planned 372 (56%) patients to 281 (42%) effectively transferred and discharged patients. The accuracy regarding disposition of patients with acute morbidity increased from 53% to 68% after observation.

**Conclusion:**

Disposition planning in patients with NSC improves after observation, if defined by the accuracy regarding hospitalization of patients with acute morbidity. Further research should focus on risk stratification tools for timely disposition planning in order to reduce high admission rates for patients without acute morbidity and high readmission rates for discharged patients with non-specific complaints.

## Introduction

Emergency departments (EDs) in Europe and North America have to cope with a continuous increase in visits [1]–[3]. Whether the ageing population is the reason for ED overcrowding is an ongoing discussion [4]–[7].

Compared to younger patients, a higher proportion of patients older than 65 years are arriving by ambulance, which may mirror a higher acuity level of their visits [1], [5]. Elderly patients have a higher likelihood of significant pathology, higher hospitalization rates, and extended lengths of stay [5], [8]. After discharge, a considerable proportion of these patients suffer from functional decline, a reduction in health-related quality of life, an increase in the use of health services, and mortality [8]–[10].

A contributing factor may be that up to 20 percent of elderly patients present to the ED with non-specific complaints (NSC) such as “not feeling well”, “feeling weak”, or were referred for lacking community support [11]–[13]. NSC have been shown to be associated with a high risk of hospital admission [14].

It was shown that the differential diagnosis of NSC is extremely broad and that there is a serious underlying condition in 60% [15], [16]. Therefore, an early and safe disposition of patients with NSC by an optimal determination of the level of care, ranging from discharge to intensive care, is crucial.

The objective of this study was to prospectively study the disposition process of patients presenting to the ED with NSC. Furthermore, our aim was to evaluate the disposition of patients with NSC in terms of adequacy of discharge, transfer, and admission.

## Methods

The wording of this manuscript is suitable for publication.

### Study Design

The Basel Non-Specific Complaints (BANC) study is a prospective, observational delayed type cross-sectional diagnostic study with a 30-day follow-up. The study was conducted in the ED of the University Hospital of Basel, Switzerland. In this urban 700-bed university hospital serving a population of 500,000, more than 45,000 trauma and non-trauma patients are seen in the ED every year, whereat elderly patients account for about 20% of all visits. The overall admission rate is 29.1%, 55% for the elderly population. The community has three geriatric hospitals, two hospices for palliative care, and 39 nursing homes with almost 2,900 nursing beds. Homecare is provided for 6,700 patients.

We confirm that the study has been approved by (EKBB 73/07), an institutional ethics committee. EKBB: Ethik Kommission Beider Basel. Written consent was obtained. The BANC study is registered with ClincalTrials.gov number NCT00920491 and is in compliance with the Helsinki Declaration.

### Inclusion Criteria

From May 24^th^ 2007 until May 14^th^ 2009 all consecutive non-trauma patients with an Emergency Severity Index (ESI) of 2 or 3 presenting to the ED were screened for inclusion [17]. The ESI (including its German version) has been proven to be a triage tool with excellent inter-rater reliability and validity, but with a slightly reduced performance in the elderly population [17], [18].

Patients qualified for inclusion if they presented with non-specific complaints to the ED (see definition below and previous description [15]).

In contrast, patients presenting with specific complaints (e.g. abdominal pain) were not considered for inclusion, since their work-up is usually protocol-based or straight-forward. If patients were hemodynamically unstable, showed persistent signs of shock, or presented with vital parameters significantly out of normal range (systolic blood pressure <90 mm Hg, heart rate >120 beats/min, temperature >38.4°C, respiratory rate >30 breaths/min, SaO_2_<92%) they were not included in the study.

### Data Collection

The following patient data were obtained by a study physician during ED evaluation: Demographic baseline data, presenting complaints, vital signs, medical history, physical examination, Katz Activities of Daily Living (ADL) [19], [20], interpretation of routine ECG, comorbidities according to the Charlson comorbidity index (CCI) [21] and prescribed drugs were collected from patient history, physician reports and patient charts.

Furthermore, for each study patient, the scores of the *“Identification of Seniors at Risk (ISAR)”* and the *“Triage Risk Screening Tool (TRST)”* were calculated [22]–[24]. The ISAR scale consists of six questions about previous hospitalization, medication use, functional impairment, and need for help before and after hospital discharge. It has been validated for patients discharged from the ED and showed an acceptable to excellent predictive validity for a variety of outcomes, such as mortality, functional decline, readmission, and institutionalization. [22], [23].

The TRST identifies baseline functional impairment in older ED patients and is moderately predictive of subsequent functional decline after an initial ED visit [24], [25].

All patients taking part in the BANC study were evaluated and treated at the discretion of the ED physician in charge, a senior physician, board-certified in internal medicine, present for 24 hours on 365 days. No intervention was performed.

### Definitions

#### Non-Specific Complaints (NSC)

We defined NSCs as all complaints that are not part of the set of specific complaints or signs or where an initial working diagnosis cannot be definitively established. In contrast to NSC, a specific chief complaint usually provides key information that allows the generation of a working diagnosis and the initiation of a predefined diagnostic and/or treatment protocol. Specific complaints are well-recognized as such in the literature, and diagnostic protocols are often applied [26].

It is necessary to define NSCs as the remainder, after exclusion of specific complaints, because an active definition would require an almost endless enumeration of possible NSCs. Common non-specific complaints are “general weakness”, “not feeling well”, “being tired” or descriptions from health care providers such as “general deterioration”, “home care impossible”, or “lack of community support” [13], [15], [27].

#### Disposition

Disposition in our institution consists of a two-step process: the first disposition-plan is made within four hours (“intention to transfer”) in the triage area. Due to persisting access blocks, elderly patients remain under observation for up to 24 hours, usually in our observation unit (OU).

The second disposition plan (“effective transfer”) is established up to 20 hours later in a standardized fashion by means of a team session led by the senior physician. At this time, information about the course of disease, follow-up lab exams and clinical data is available [12].

If acute morbidity (i.e. pneumonia, urosepsis) is identified in the triage area, admission to an inpatient acute ward is requested by the attending physician (code “A” for “acute”).

If the patient has no acute morbidity, but is in need for further geriatric assessment or not suitable for outpatient work-up, transfer to a geriatric unit or hospital is requested by the attending physician (code “G” for “geriatric”).

**Figure 1:**
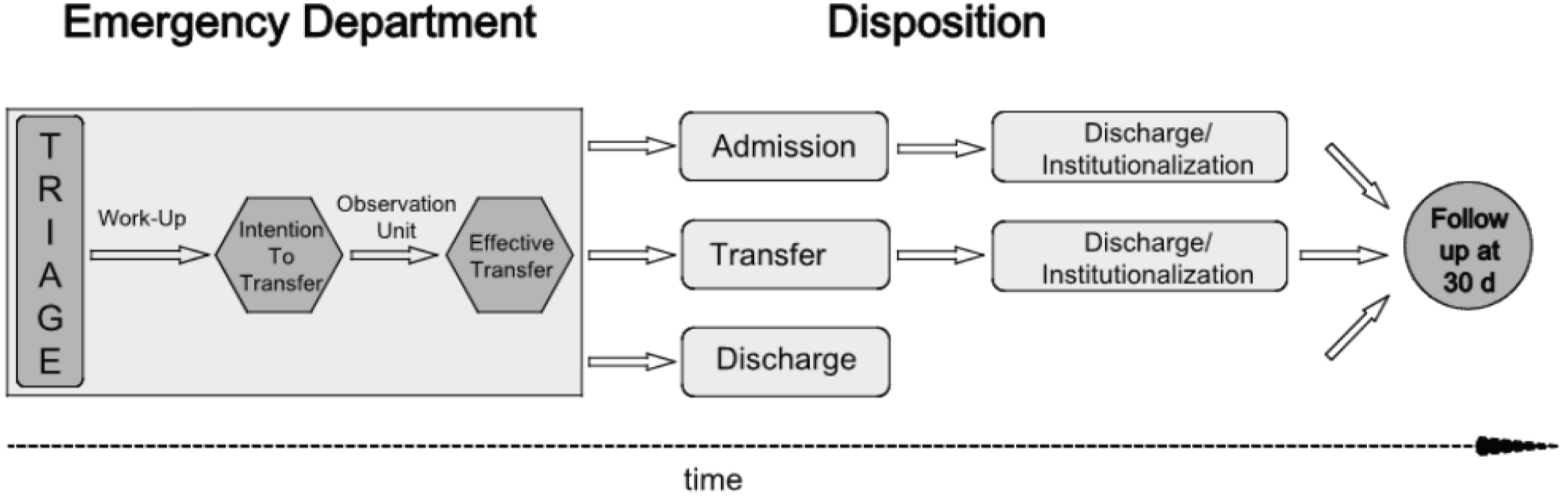
Disposition process of patients with non-specific complaints as taking place in the ED of university hospital Basel, Switzerland.

All patients suitable for outpatient work-up are discharged by the attending physician (code “D” for discharge). See Figure 1.

#### Definition appropriate versus inappropriate Disposition

Appropriate Disposition is defined as transfer of patients with acute morbidity to an acute-care ward (e.g. internal medicine), or, in patients without acute morbidity, either transfer to a geriatric hospital or discharge from the ED.

Accuracy of disposition in our study setting is defined as the proportion of patients with acute morbidity which are correctly identified as such by being admitted to an acute ward (number of admitted patients with acute morbidity (true positive rate), divided by the sum of the number of true positives (admission of patients with acute morbidity), and false negatives (transfer and discharge of patients with acute morbidity)).

### Patient Follow-up and Outcome Ascertainment

The follow-up period was 30 days [15]. All hospital and discharge reports were obtained, and family physicians were contacted in cases discharged before 30 days.

All outcome data were reviewed by two outcome assessors (two senior emergency physicians, board certified in internal medicine and a subspecialty) and a final diagnosis related to the initial complaints was established according to the 10^th^ International Classification of Diseases and Related Health Problems (ICD-10). Additionally, they decided about the presence of acute morbidity (serious condition) for the time of ED presentation. Acute morbidity (serious condition) was defined as any life-threatening condition or any condition requiring early intervention (within 24 hours) to prevent health status deterioration according to a predefined list [15]. Any death occurring within 30 days of the initial ED presentation was also defined as serious condition.

Further outcome data, such as rehospitalization within 30 days, were obtained. The total length of stay (non-interrupted hospitalization) was recorded.

## Results

Between May 24^th^ 2007 and May 14^th^ 2009, a total of 22,782 patients presenting to the Emergency Department were screened for inclusion. Among 9926 non-trauma patients, 9212 patients presented with specific complaints (the most frequent being chest pain, dyspnea, abdominal pain, syncope, stroke-like symptoms, and nausea with vomiting) and therefore were excluded. The final study population consisted of 686 patients presenting to the ED with NSC. In 17 cases, which all have been admitted, the primary disposition plan was not recorded and therefore couldn’t been analyzed. A total of 669 patient records remained for analyzes. Median age was 81 (IQR 72/87) years, 62% were female, 574 (85.8%) were older than 64 years. Study subjects had a median of four comorbidities, and took a median of five different medications daily. The median Charlson Comorbidity Index [21] was two. More than half (52%) of all patients with NSC had been hospitalized during the previous year, and 9% were living in a nursing home. 515 patients (77%) were independent in at least 4 out of 6 ADL according to Katz. 97% of all subjects aged 65 years and older had an increased risk for adverse outcomes such as death, functional decline, institutionalization, and readmission according to the ISAR score [22]. According to the TRST score [24], 99% of all patients older than 64 years were at high risk for hospitalization, institutionalization in nursing homes, or readmission to ED (Table 1).

**Table 1:** Baseline Demographic Characteristics of the BANC Population.

Characteristic	No	%
Total No.	669	
Male subjects	254	38
Age, y – Median (IQR)	81 (72/87)	
Age 65–84 y	341	51
Age ≥85 y	233	34.8
ESI score		
2	23	3.4
3	646	96.6
Current Comorbidities, Median (IQR)	4 (3/6)	
Charlson Comorbidity Index, Median (IQR)	2 (1/4)	
Current medications, Median (IQR)	5 (3/8)	
Activities of daily living performed, Median (IQR)	6 (4/6)	
4–6	515	77
≤3	154	23
ED visit previous year	200	29.9
Hospitalizations during previous year	357	52
Institutionalized in nursing home at index visit	62	9.3
ISAR at risk (patients at age ≥65)	445	97
TRST at risk (patients at age ≥65)	537	99

(IQR = Interquartile Range, y = years, ESI score = Emergency Severity Index score, ED = Emergency department, ISAR = Identification of Seniors At Risk, TRST = Triage Risk Screening Tool).

### First Disposition Plan: Intention To Transfer (ITT)

After initial work-up in the triage area, 297 (44%) of the 669 patients were planned for admission to an acute care ward, 278 (42%) patients were planned for transfer to a geriatric hospital, and 94 (14%) patients were to be discharged.

### Final Disposition: Effective Transfer (ET)

After the observation period, disposition was adapted as follows: 388 (58%) were admitted to an acute care ward, and 190 (28%) patients were transferred to a geriatric community hospital. 91 patients (14%) were discharged.

### Modification of Disposition Plan in Patients with Acute Morbidity

**Figure 2:**
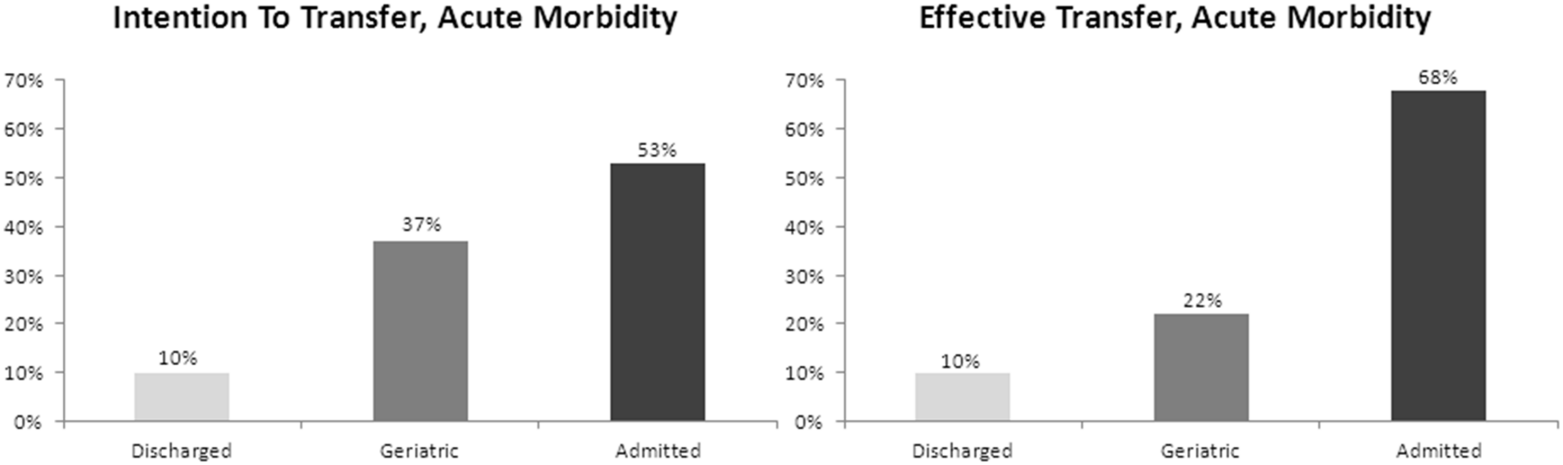
Modification of disposition plan in patients with acute morbidity after observation time of up to 24 hours, shown for discharged, transferred to geriatric hospital and admitted patients.

In this study, 399 (60%) out of 669 patients were attributed a *serious condition,* and the number of “A” patients in this group increased from 211 to 270 (52.9% to 67.7%), while the number of “G” and “D” patients decreased from 188 to 129 (47.1% to 32.3%) after observation (figure 2).

Accuracy of “optimal disposition” was 53% at the first disposition decision (ITT), and increased to 68% at the second disposition decision (ET).

### Length of Stay

**Figure 3:**
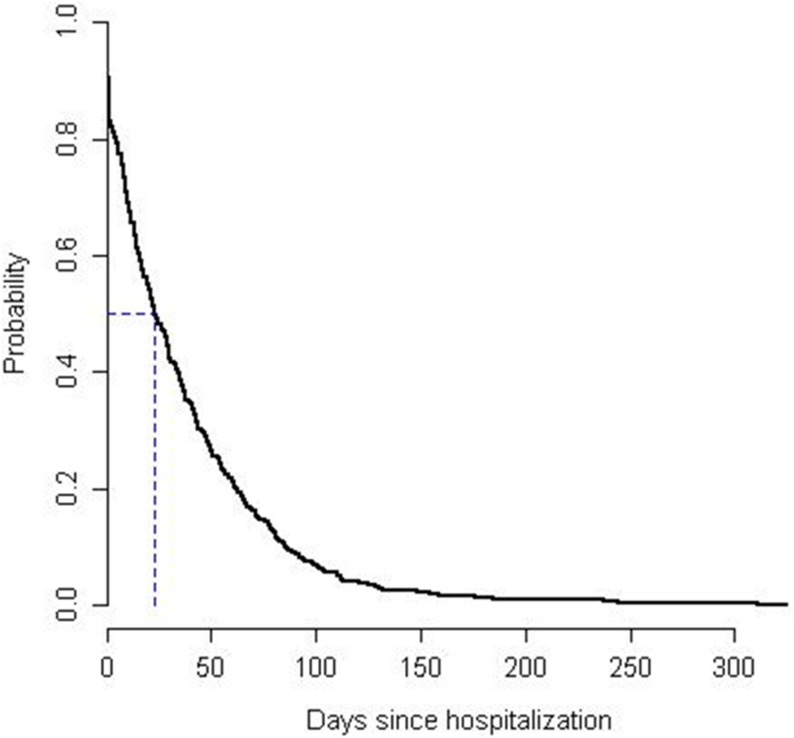
Kaplan-Meier-Curve for hospital length of stay in the BANC study population (n = 669).

Patients were hospitalized for an average of 36.7 days (SD 41.7 days, Median 23 days) with a longest stay of 325 days. Any patient presenting to the ED with non-specific complaints and an ESI triage score of 2 or 3 had a probability of 45% to be hospitalized for at least 30 days (figure 3). Death within 30 days occurred in 40 (6%) of all study patients. More deaths occurred in patients admitted to an acute ward (A) than in geriatric patients (G) (Table 2).

**Table 2:** Characteristics of the BANC study population, shown for admitted, transferred to geriatric hospital and discharged patients.

Characteristic	A	G	D
Number in group (%)	388 (58)	190 (28)	91 (14)
Male Subjects (%)	159 (41)	57 (30)	38 (42)
Age, Median (IQR)	79.5 (71/86)	85 (79/89)	75 (58.5/82.5)
Total LOS [days], Median (IQR)	28.5 (12/56.5)	37.5 (19/61)	–
Survivors			
LOS ≥14 days, N (%)	253 (70%)	148 (81%)	–
*Serious*	*168 (69%)*	*77 (87%)*	*–*
*Non-serious*	*85 (72%)*	*71 (76%)*	*–*
LOS ≥30 days, N (%)	*186 (51%)*	*111 (61%)*	*–*
*Serious*	*124 (51%)*	*60 (58%)*	–
*Non-serious*	*62 (52%)*	*51 (54%)*	*–*
Death within 30 days, N (%)	28 (7.2)	9 (4.7)	3 (3.3)

(LOS = length of stay, IQR = Interquartile Range, “A” = Admission, “G” = Transfer to Geriatric hospital, “D” = Discharge).

In survivors, 70% of the patients admitted to acute ward (A) and 81% of the geriatric patients (G) had a LOS more than 14 days. And by the end of the follow up period of 30 days, half of the patients admitted (A) and 61% of the geriatric patients (G) were still hospitalised.

## Discussion

The purpose of this study was to prospectively study the disposition process of patients with non-specific complaints. Our results show that the accuracy of adequate disposition of patients with acute morbidity increases after observation. On the other hand, disposition of patients presenting to the ED with non-specific complaints remains insufficient, even after an overnight stay. The first disposition plan (after the initial work-up) was almost a matter of chance. Whereas 68% of all patients with acute morbidity were finally admitted to an acute care ward, the remaining 32% were transferred to a geriatric hospital, or were discharged. This improvement can be interpreted as an advantage of a prolonged work-up or the presence of an observation unit. However, the higher accuracy regarding hospitalization of patients with acute morbidity may also be attributed to the fact that for all patients on the observation unit the team session was decisive for the ultimate disposition plan, and could therefore be attributed to the “wisdom of the crowd” effect.

These difficulties of disposition planning need to be discussed in the context of other challenges in elderly patients with NSC. Their work-up has not been standardized, and might therefore differ largely between different health-care systems, and even between individual emergency physicians.

The obvious challenge in diagnosing elderly patients with NSC could be addressed in two ways: either by identifying patients at risk of acute morbidity quicker (including the opportunity to rapidly admit them to an acute care ward), or by identifying patients with rehab potential (who should be quickly transferred to geriatric hospitals/wards). Given the lack of time and the increasing workload in the ED, thoroughly evaluating every patient older than 65 years with a comprehensive geriatric assessment is not realistic. Therefore, attempts should aim at identifying “high-risk” elderly patients [28]. To date, there are several scores to estimate the risk for adverse outcomes, such as death, functional decline, institutionalization, and readmission in patients older than 65 years. However, their clinical usefulness is controversial [24], [29]. According to the ISAR and TRST score respectively, almost all patients older than 64 years in our study population were at risk for adverse outcomes (Table 1). Therefore, these scores do not seem to provide helpful additional information regarding the optimal disposition planning in patients presenting with non-specific complaints. Hence, new stress-biomarkers may be a useful risk stratification tool to support disposition planning in frail elderly patients. They have shown to predict mortality in patients presenting to the emergency department with nonspecific complaints [30], [31].

While finding the correct diagnoses in this patient population can take hours to several days and overwhelming outpatient work-up [32] and growing requirements for accurate and quick decisions force emergency physicians to walk the tight rope, an observation unit could indeed be a tool for improving disposition in this patient group. The impact of a standardized team session should also be taken into account as an effect towards a higher accuracy of later disposition decisions, as well as a prolonged observation by nurses that may be a key factor for optimal disposition.

In order to distinguish the 60% with a serious underlying condition from the 40% with no acute morbidity at the earliest possible point of time, risk stratification needs to be implemented – possibly even before diagnoses are made – because i) transfer of patients with an acute morbidity to a geriatric hospital may lead to a higher back-transfer rate and ii) discharge of patients with acute morbidity may increase readmission – in our study, 34% rehospitalization rate in discharged versus 3% in admitted patients. However, these results are well in accordance with the literature, where elderly patients were shown to be at increased risk for repeated ED visits and hospitalizations [32] even after observation [33]. Furthermore, elderly patients are more likely to be misdiagnosed and, consequently, are more frequently discharged with unrecognized and untreated health problems, which is confirmed by our own results [8].

We included the analyses of length of stay (LOS) in this paper since it reflects the general morbidity and frailty of our patient population. The majority of admitted patients had a LOS of more than 14 days, independent of their disposition or the acuity of their condition. In times where a lot of health systems work with diagnosis related groups (DRG) and struggle with cost effective patient management minimizing the LOS in acute hospital wards is crucial. Our analysis reflects the vulnerability of frail patients, in whom even a minor illness can lead to a functional decline and whose recovery times are much longer than in healthy independent individuals. Therefore, an early distinction of those patients with acute morbidity and their adequate disposition is not only crucial for their return to baseline homeostasis, but also for cost effectiveness [34].

Recently it has been shown that the use of observation may increase patient safety and satisfaction while decreasing unnecessary inpatient admissions [35]. The latter is as important as selecting the patients with acute morbidity for admission to an acute care ward. In our study population, patients without acute morbidity most often require a comprehensive geriatric assessment. The advantages of geriatric hospitals compared to an acute medical ward are lower costs, and higher expertise in geriatric assessment and adequate early rehab measures.

However, most EDs do not have the possibility to observe patients for up to 24 hours in order to make better diagnoses and dispositions: It has been estimated that only about a third of all EDs have an observation unit [36].

### Limitations

Because this study was performed at a single urban tertiary care center serving Northwestern Switzerland, the external validity is limited.

Furthermore, the university hospital of Basel has the opportunity to transfer patients to geriatric community hospitals. This disposition may be not available to other EDs.

While patients were observed in our observation unit for up to 24 hours, the actual cause for the non-specific presentation may have slowly unraveled. Since many EDs lack this type of observation unit, the results of this study cannot be generalized.

## Conclusion

This observational study shows that patients with NSC are at high risk for inappropriate disposition planning. This may add to the increased risk for adverse health outcomes. The accuracy regarding hospitalization of patients with acute and serious conditions increases after observation, suggesting a potential benefit of an observation unit.
